# Understanding longitudinal biventricular structural and functional changes in a pulmonary hypertension Sugen–hypoxia rat model by cardiac magnetic resonance imaging

**DOI:** 10.1177/2045894019897513

**Published:** 2020-02-10

**Authors:** Geeshath Jayasekera, Kathryn S. Wilson, Hanna Buist, Rosemary Woodward, Aysel Uckan, Colin Hughes, Margaret Nilsen, A Colin Church, Martin K. Johnson, Lindsay Gallagher, James Mullin, Mandy R. MacLean, William M. Holmes, Andrew J. Peacock, David J. Welsh

**Affiliations:** 1 ICAMS, University of Glasgow, Glasgow, UK; 2 Scottish Pulmonary Vascular Unit, Glasgow Caledonian University, Glasgow, UK; 3 CRF, University of Glasgow, Glasgow, UK; 4 GEMRIC, INP, University of Glasgow, Glasgow, UK

**Keywords:** pulmonary arterial hypertension, cardiac magnetic resonance (CMR), right ventricle, left ventricle

## Abstract

Cardiac magnetic resonance-derived ventricular variables are predictive of mortality in pulmonary arterial hypertension. Rodent models which emphasize ventricular function, allowing serial monitoring, are needed to identify pathophysiological features and novel therapies for pulmonary arterial hypertension. We investigated longitudinal changes in the Sugen–hypoxia model during disease progression. Sprague Dawley rats (*n* = 32) were divided into two groups. (1) Sugen–hypoxia: a dose of subcutaneous Sugen-5416 and placed in hypobaric hypoxia for two weeks followed by normoxia for three weeks. (2) Normoxia: maintained at normal pressure for five weeks. Rats were examined at five or eight weeks with right-heart catheter, cardiac magnetic resonance, and autopsy. Compared to normoxic controls (23.9 ± 4.1 mmHg), right ventricular systolic pressure was elevated in Sugen–hypoxia rats at five and eight weeks (40.9 ± 15.5 mmHg, *p* = 0.026; 48.9 ± 9.6 mmHg, *p* = 0.002). Right ventricular end-systolic volume index was increased in eight weeks Sugen–hypoxia (0.28 ± 0.04 µlcm^–2^, *p* = 0.003) compared to normoxic controls (0.18 ±0.03 mlcm^–2^). There was progressive dilatation of the right ventricular at eight weeks Sugen–hypoxia compared to normoxic controls (0.75 ± 0.13 µlcm^–2^ vs 0.56 ± 0.1 µlcm^–2^ *p* = 0.02). Ventricle mass index by cardiac magnetic resonance at five weeks (0.34 ± 0.06, *p* = 0.003) and eight weeks Sugen–hypoxia (0.34 ± 0.06, *p* = 0.002) were higher than normoxic controls (0.21 ± 0.04). Stroke volume, right ventricular ejection fraction, and left ventricular variables were preserved in Sugen–hypoxia. Ventricular changes during the course of illness in a pulmonary arterial hypertension rodent model can be examined by cardiac magnetic resonance. These changes including right ventricular hypertrophy and subsequent dilatation are similar to those seen in pulmonary arterial hypertension patients. Despite the persisting pulmonary hypertension, there are features of adaptive cardiac remodeling through the study duration.

## Introduction

Pulmonary arterial hypertension (PAH) is a disease of the pulmonary vasculature; however, it is subsequent right ventricular (RV) failure that is the main cause of morbidity and mortality in patients. Cardiac magnetic resonance (CMR) is a non-invasive imaging tool providing high-resolution three-dimensional images of the heart. During CMR, ventricular short axis stacks are used to reconstruct a 3D image of the right and the left ventricle (LV), and ventricular volumes, mass, and function can be measured.^1^ Many CMR measurements have shown to be strongly predictive of mortality and survival thus offering potential for assessing response to treatment. Stroke volume,^2^ RV ejection fraction,^3^ and RV and LV end-diastolic volume (EDV)^4^ have all shown to be prognostic markers in PAH patients.

Small animal (rodent) models are increasingly used to identify pathophysiology as well as therapies for PAH with the intention of translating findings to humans. Accurate monitoring of disease in rodents with emphasis on ventricular function (rather than right heart catheterization alone) without killing the animal is needed.

Various rodent models to recapitulate human PAH have been produced. Sugen-5416 (Sugen), an inhibitor of vascular endothelial growth factor was shown to cause a mild rise in pulmonary artery pressure in wild-type rats. Combining Sugen with another stimulus of pulmonary hypertension (PH), i.e. chronic hypoxia, Taraseviciene-Stewart et al. described the Sugen–hypoxia (SuHx) rat model in 2001.^5^ A combination of Sugen and chronic hypoxia cause pulmonary endothelial cell death and severe PH. Subsequently in 2010, Abe et al. showed that SuHx rats demonstrated evidence of severe pulmonary arteriopathy including concentric neo-intimal and complex plexiform-like lesions which closely resemble plexiform lesions seen in humans.^6^ Subsequently, other groups have attempted to characterize hemodynamics in a SuHx model beyond right heart catheterization alone. Vitali et al. evaluated longitudinal changes in a SuHx mouse model of PH. Echocardiographic and invasive measurements were performed after three weeks of hypoxia and after 10 weeks of recovery in normoxia. Ten weeks after hypoxic exposure, RV systolic pressure (RVSP) had decreased, but remained elevated compared to normoxic controls. However, RV hypertrophy had resolved. They observed very few angio-obliterative lesions at 10 weeks.^7^ De Raaf et al. used telemetry to characterize hemodynamics in SuHx rats and associated these with serial histology.^8^ Jones et al. demonstrated a good correlation between M-mode and Doppler Echo vs right heart catheterization in the monocrotaline rat model.^9^ In a study by Urboniene et al. assessing validation of high resolution echocardiography and CMR vs high fidelity catheterization in experimental PH monocrotaline rat model, non-invasive measures of RV free wall thickness/mass correlated well with post mortem measurements.^10^

Our group has a proven track record using CMR imaging to evaluate RV function in humans with PAH.^4,11,12^ The same non-invasive and repeatable measurements would be of great advantage for the study of rodent models to allow a detailed understanding of ventricular structural and functional changes that occur, to enhance efficacy in translational medicine. We investigated whether CMR is feasible in a SuHx rat model of PH. Subsequently, we investigated the structural and functional changes associated with the model during disease progression. Finally, we discussed the suitability of the SuHx model for translational studies of the mechanisms of RV dysfunction in PAH.

## Methods

### Ethics

All experimental procedures were carried out in accordance with the United Kingdom Animal Procedures Act (1986) and with the US NIH publication No. 85-23, revised 1996, and ethical approval was also granted by the University of Glasgow Ethics Committee. Rodents were housed in a 12-h light–dark cycle with access to food and water ad libitum.

### Study design

**Fig. 1.**
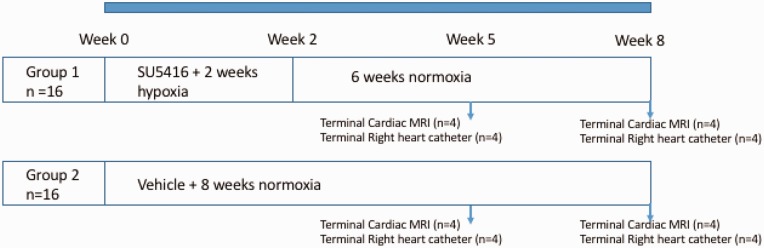
The study design summarized. Male Sprague Dawley rats (*n* = 32) were divided into two groups (*n* = 16 in each group). Group 1: Sugen–hypoxia (SuHx)—a single dose of Sugen-5416, before being placed in a hypobaric chamber for two weeks and then placed in normal room pressure for three weeks whilst PH developed, animals receive control chow throughout. Group 2: normoxic maintained at normal room pressure for five weeks. In each group, half the animals entered the cardiac MRI arm of the study (*n* = 8) while the other half underwent right heart catheterization for hemodynamic assessment (*n* = 8). Animals were assessed at five weeks and eight weeks from the beginning of the study with right heart catheterization, CMR, and gross anatomy at autopsy.

Male Sprague Dawley rats (three weeks) (*n* = 32) were divided into two groups (*n* = 16 in each group). Group 1: *SuHx*—a single dose of subcutaneous Sugen-5416 (Sigma, UK) suspended in vehicle (20 mg/kg), before being placed in a hypobaric chamber (atmospheric pressure 550 mbar) for two weeks and then placed in normal room pressure (1013 mbar) for three weeks whilst PH developed. Group 2: *Normoxia*—maintained at normal room pressure for five weeks. In each group (*n* = 16), half the animals entered the CMR arm of the study (*n* = 8) while the other half underwent right heart catheterization for hemodynamic assessment (*n* = 8). Animals were assessed at five weeks and eight weeks from the beginning of the study. The study design is summarized in Fig. 1.

### In vivo hemodynamic measurements

Animals were anesthetically induced with 3% (v/v) isoflurane and then maintained at 2% (v/v) isoflurane supplemented with a constant flow of 5% (v/v) oxygen. Hemodynamic measurements were taken using an ultra-miniature Polyimide Nylon catheter capable of measuring ventricular pressure continuously (AD Instruments SPR-869NR, Millar). The catheter was used as per the manufacturer’s instructions with the PowerLab 35 Series data acquisition system with LabChart Pro and the pressure volume (PV) loop analysis module. For right heart pressure analysis, the catheter was inserted into the jugular vein and guided into the RV to measure RVSP.

### RV hypertrophy and tissue harvest

Following hemodynamic assessment, animals were culled and hearts flushed with phosphate buffered saline using a blunt needle to clear peripheral blood cells. Euthanasia consisted of an overdose of anesthetic followed by a schedule 1 kill (cervical dislocation). The heart was isolated, atria removed, and tissue fixed with 10% (v/v) neutral buffered formalin for 48 h before paraffin processing for histological analysis by immunohistochemistry. Tissue was sectioned by microtome at width of 5 µm and stained by Gomori’s trichrome staining kit (Atom Scientific, Manchester, UK) as per manufacturer’s instructions.

### Gross anatomy postpartum

After the heart was isolated and atria removed, the RV and LV weights were obtained to determine RV hypertrophy. Interventricular septum was considered part of the LV.

### Pulmonary vascular remodeling

Vascular thickening was determined by smooth muscle actin antibody (ab5694, Abcam, Cambridge, UK) staining, thickening was characterized by an increase in the vessel wall diameter of more than 50% of the arterial wall or complete occlusion. The number of remodeled vessels over the total number of vessels present in a lung section was determined. Sections were analyzed in a blinded manner.

### CMR

CMR imaging was performed in a Bruker Biospec 7-T/30-cm (Bruker Biospin, Ettlingen, Germany) system with a gradient coil insert (400 mT/m). Using a 72-mm transmit birdcage resonator and four channel phased array rat cardiac receiver coil. Anesthesia was induced with gas flow at 2–3 l/min, and the isoflurane delivered via a vaporizer (Vetamac, Rossville, IN) at 3–4%. The exhaust was connected to the Omnicon F/Air device (AM Bickford). After induction, animals were maintained at 2% (v/v) isoflurane supplemented with a constant flow of 5% (v/v) oxygen. An external water jacket was used to maintain a core temperature of 37℃. During all procedures, body temperature, ECG, and respiration were monitored (Echo: Indus Instruments, Houston, TX; MRI: SA Instruments, Stony Brook, NY; Cath: Powerlab, AdInstruments, Colorado Springs, CO). Long and short axis scout images were acquired so that short axis images could be planned using a segmented, cardiac-triggered FLASH sequence. The images were acquired with a slice thickness of 1.5 mm, ensuring the entire biventricular length is covered. The CMR parameters were as follows. Slice thickness—1.50 mm, field of view—30.00 mm × 30.00 mm, image matrix—192 × 192 pixels, image resolution—156 µm × 156 µm, Flip angle—15 degrees, Echo time—2.50 ms, Rep. time—7.02 ms, number of frames—25, number of averages—6, and software version—Paravision 5.1.

### CMR analysis

Scans were coded by number and analyzed in batches by G.J who was blinded to the identity and hemodynamic results at the time of analysis. A second observer (A.U) analyzed five scans for inter-rater agreement analysis. Trabeculations and papillary muscles were considered as part of the blood pool. The epicardial and endocardial borders were manually outlined in end-diastolic and end-systolic frames using QMass (MEDIS, Netherlands). Stroke volume was determined from EDV – end-systolic volume (ESV) of the LV. Ejection fraction ((SV/EDV) × 100%) was also determined. RV and LV masses were determined by manual planimetry at diastole. Ventricular mass index (VMI) was defined as the ratio between RV to LV mass, with the interventricular septum considered part of the LV. LV eccentricity index (LVEI) was defined as the ratio between maximum anterior–posterior to septal lateral diameters of the LV and was measured at both systole and diastole. All ventricular volumes and mass measurements were indexed to body surface area.^13^

### Statistical analysis

Ventricular volumes and mass are given as µlcm^–2^ and mgcm^–2^, respectively, indexed for body surface area. Statistical analysis was performed using SPSS (IBM, SPSS Statistics, USA) and GraphPad Prism (GraphPad, USA). A significance level of 0.05 was employed for statistical tests. An analysis of variance test was used to compare RV and LV mass, volumes, and function between normoxic animals and different stages of SuHx. If there was statistical significance, a Tukey test was used for post hoc analysis. To compare different methods of VMI measurement (CMR vs autopsy), a spearmen correlation was used. Inter-rater variability for determination of LV and RV function were calculated from paired measurements of the LV ejection fraction (LVEF) and RV ejection fraction (RVEF) of two readers as interclass correlation coefficient with a two-way mixed model for absolute agreement. Results are shown as mean ± SD unless otherwise stated.

## Results

### Right heart catheterization and RV hypertrophy

**Fig. 2.**
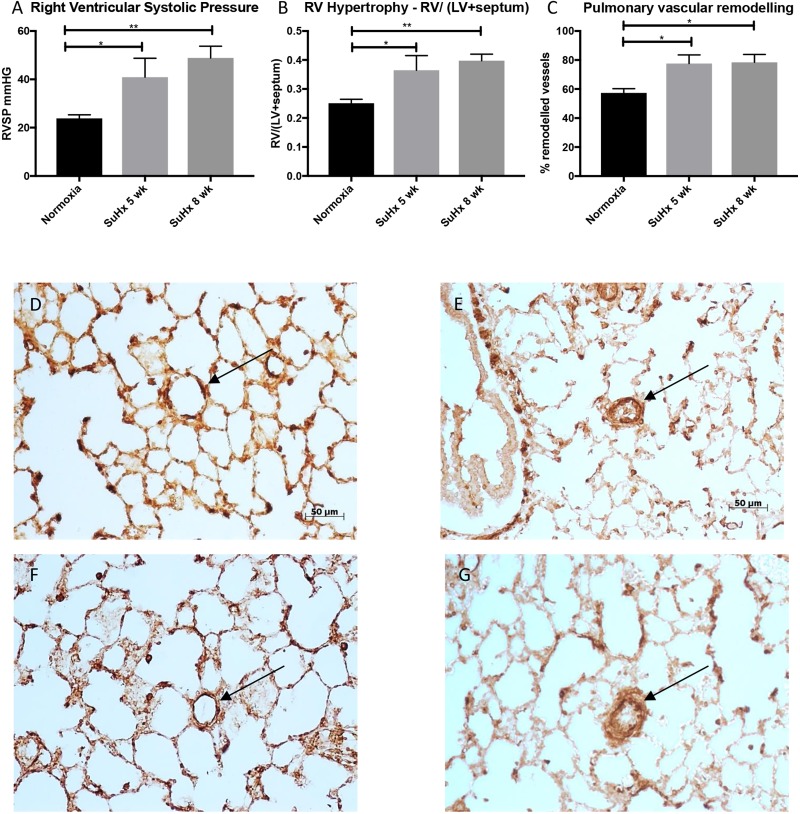
Right ventricular systolic pressure measured by right heart catheterization (a), RV/(LV + septum) by gross weight (b), the percentage of remodeled vessels in a lung section (c), and immunohistochemical analysis of α-smooth muscle actin (α-SMA) staining in the smooth muscle layer of small pulmonary arteries of the lungs ((d) to (g)). For right heart pressure analysis, the catheter was inserted into the jugular vein and guided into the right ventricle to measure right ventricular systolic pressure (RVSP). Animals were culled, the heart was isolated, atria were removed and RV/(LV + septum) were measured to assess RV hypertrophy. Although there are significant differences between the normoxic group (*n* = 8) and Sugen–hypoxic groups at five (*n* = 4) and eight weeks (*n* = 4), there were no significant differences in RVSP nor RV/(LV + septum) at autopsy between Sugen–hypoxia five and eight weeks. Vascular thickening was determined by smooth muscle actin antibody (ab5694, Abcam, Cambridge, UK) staining, thickening was characterized by an increase in the vessel wall diameter of more than 50% of the arterial wall or complete occlusion. The number of remodeled vessels over the total number of vessels present in a lung section was determined. Results are shown as mean ± SEM. ANOVA test was used to compare the three groups, and if there was statistical significance, a Tukey HSD test was used for post hoc analysis. * represents *p* < 0.05 and ** represents *p* < 0.01. For immunohistochemical analysis of α-smooth muscle actin (α-SMA) staining in the smooth muscle layer of small pulmonary arteries of the lung, sections were viewed at ×200. (d) and (f) demonstrate normoxic animals at five and eight weeks while (e) and (g) demonstrate vascular thickening and remodeling of the pulmonary vasculature (black arrows) in Sugen–hypoxic rats at five and eight weeks, respectively. RVSP: RV systolic pressure; SuHx: Sugen–hypoxia; RV: right ventricle; LV: left ventricle.

Compared to normoxic rats (23.87 ± 4.1 mmHg), RVSP was significantly elevated in SuHx rats at both five and eight weeks (40.95 ± 15.5 mmHg, *p* = 0.03; 48.89 ± 9.6 mmHg, *p* = 0.002, respectively). There were no significant differences in RVSP between SuHx rats at five and eight weeks. Similarly, relative RV mass measured at autopsy by RV/(LV + septum) was significantly elevated at five weeks (0.36 ± 0.1, *p* = 0.021) and eight weeks (0.4 ± 0.04, *p* = 0.004) SuHx compared to controls (0.25 ± 0.04) (Fig. 2). Immunohistochemical analysis of α-smooth muscle actin staining in the smooth muscle layer of small pulmonary arteries of the lungs demonstrated vascular thickening and remodeling at both five and eight weeks of SuHx. Although there was statistical significant difference between the percentage of remodeled vessels between normoxia and SuHx groups (57.4% ± 7.3 vs 77.6% ± 10.3, *p* = 0.02; 57.4% ± 7.3 vs 78.5% ± 9.4, *p* = 0.02), there was no significant difference observed between SuHx rats at five and eight weeks (Fig. 2).

### CMR

**Fig. 3.**
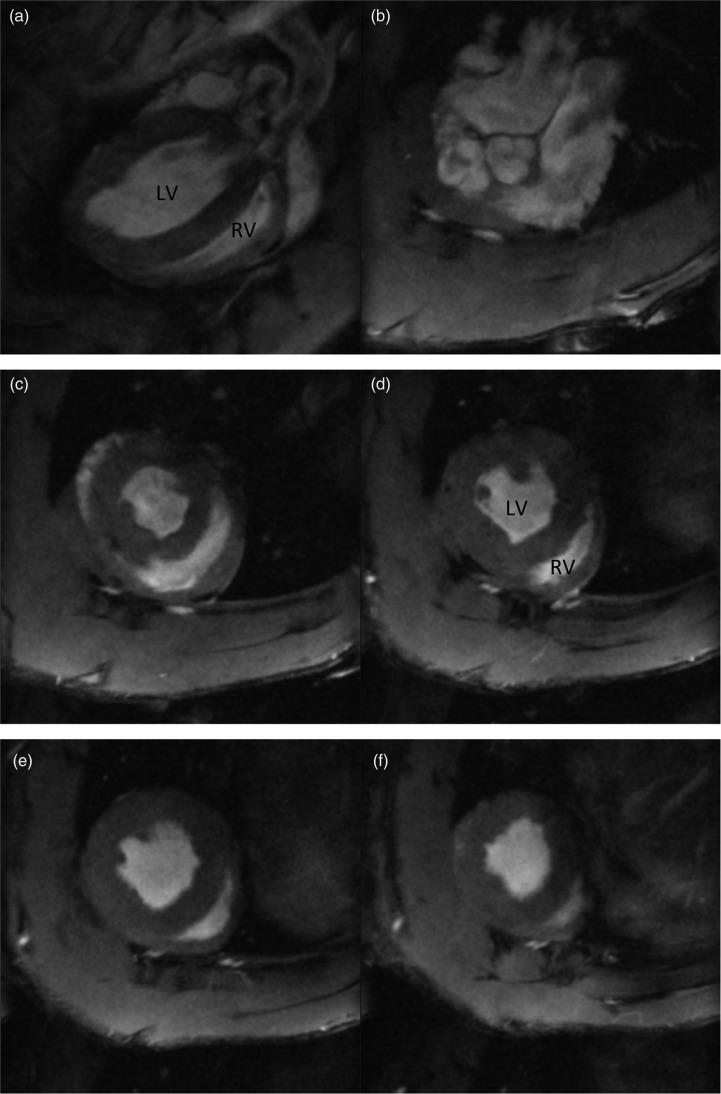
Cardiac MRI short axis image taken from a long axis (a) and a short axis cine stack ((b) to (f)) of a normoxic Sprague Dawley rat. CMR imaging was performed in a Bruker 7T (Bruker, Biospin, Germany) system. Anesthesia was induced by placing the rat in an anesthesia induction chamber with gas flow at 2–3 l/min, and the isoflurane was delivered via a vaporizer (Vetamac, Rossville, IN) at 3–4%. After induction, animals were then maintained at 2% (v/v) isoflurane supplemented with a constant flow of 5% (v/v) oxygen. An external water jacket was used to maintain a core temperature of 37℃. During all procedures, body temperature, ECG, and respiration were monitored using a rodent monitoring system (Echo: Indus Instruments, Houston, TX; MRI: SA Instruments, Stony Brook, NY; Cath: Powerlab, Ad instruments, Colorado Springs, CO). Long axis and short axis scout images were acquired so that a true short axis images could be planned using a segmented, cardiac-triggered FLASH sequence. The short axis images were acquired with a slice thickness of 1.5 mm ensuring the entire biventricular length is covered. LV: left ventricle; RV: right ventricle.

A representative long axis image (a) and a short axis cine stack (b) to (f) are shown in Fig. 3. The LV and the RV demonstrated good spatial and temporal resolution allowing manual planimetry. All of the images in normoxic or SuHx animals were suitable for analysis. Initial scout images were acquired to identify the cardiac chambers.

### Inter-rater variability

There was excellent agreement between the two observers (ICC: Intra class correlation coefficient 0.97, 95%CI: 0.74–1.0) for LVEF as well as for RVEF (ICC: 0.96, 95%CI: 0.64–1.0).

### Right ventricle

**Fig. 4.**
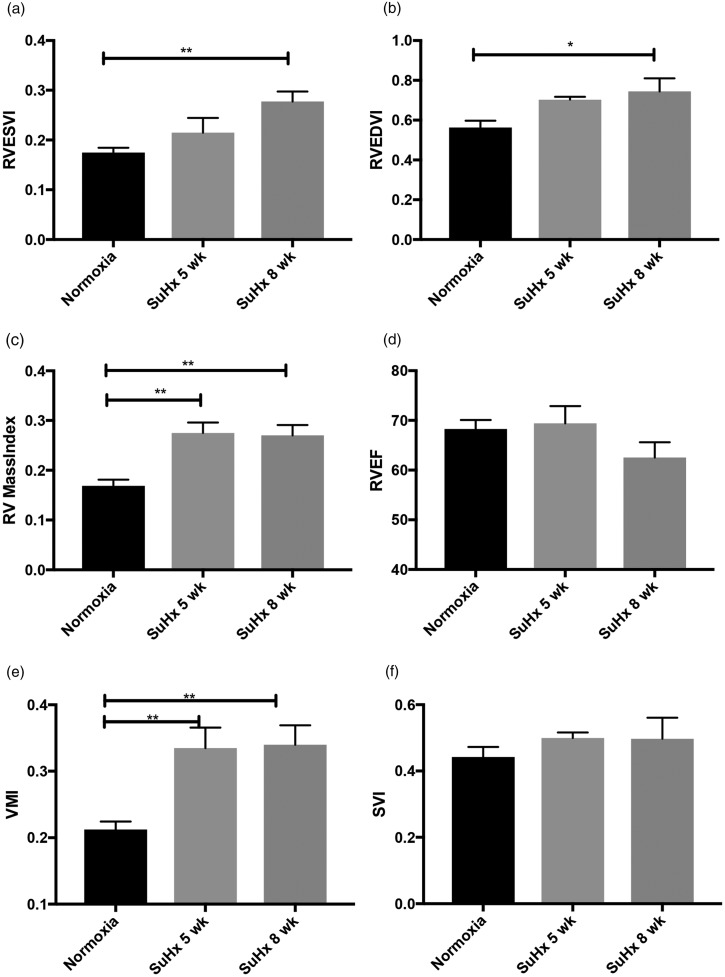
RV end-systolic volume index (RVESVI) (a), RV end-diastolic volume index (RVEDVI) (b), RV mass index (c), RV ejection fraction (RVEF) (d), ventricular mass index (VMI) (e), and Stroke volume index (SVI) (f) in normoxia (*n* = 8), five week Sugen–hypoxia (*n* = 4) and eight week Sugen–hypoxia (*n* = 4). RV end-diastolic and end-systolic volumes and RV mass were determined by manual planimetry and indexed to body surface area. RVEF was determined by ((RVEDV – RVESV)/RVEDV) × 100%. A demonstrates progressive increase in RV end-systolic volume index during the course of Sugen–hypoxia, while B demonstrates RV dilatation at eight weeks. RV mass index (c) was significantly increased in five week and eight week Sugen–hypoxia compared to normoxic rats. RVEF (d) was preserved, however, trending toward deterioration at eight weeks. Ventricular mass index (VMI) was calculated as the ratio between RV mass to LV mass. Interventricular septum was considered as part of the LV. VMI maybe an alternative to RV/(LV + septum) measured at autopsy as discussed. Results demonstrated increased VMI at five weeks and eight weeks of Sugen–hypoxia compared to normoxic rats (e). There were no significant differences in stroke volume index (SVI) between normoxia, five week Sugen–hypoxia and eight week Sugen–hypoxia (f). Results are shown as mean ± SEM. The groups were compared by ANOVA and if there was statistical significance, a Tukey HSD test was used for post hoc. LV: left ventricle; RV: right ventricle; SuHx: Sugen–hypoxia.

RVESV index was significantly increased in SuHx rats at eight weeks (0.28 ± 0.04, *p* = 0.003) compared to normoxic rats (0.18 ± 0.03). There were no significant differences between normoxia and SuHx at five weeks. Compared to normoxic rats (0.17 ± 0.03), RV mass index was increased in the SuHx rats at five weeks (0.28 ± 0.04, *p* = 0.002) and eight weeks (0.27 ± 0.04, *p* = 0.002). RV demonstrated progressive dilatation (increasing RVEDV index) at eight weeks of SuHx compared to normoxic rats (0.75 ± 0.13 vs 0.56 ± 0.1, *p* = 0.022). In RVEF, there were no significant differences between normoxic rats and five and eight week SuHx rats; however, demonstrating trends toward impairment (RVEF = 68.3 ± 5.1%, 69.4 ± 6.9%, and 62.6 ± 6.1%, respectively). Stroke volume index was preserved in the SuHx model at five and eight weeks compared to normoxia (0.44 ± 0.1 vs 0.5 ± 0.04 vs 0.49 ± 0.1). VMI after five weeks (0.34 ± 0.06, *p* = 0.003) and eight weeks of SuHx (0.34 ± 0.06, *p* = 0.002) were significantly higher than normoxic rats (0.21 ± 0.04) (Fig. 4).

### Left ventricle

**Fig. 5.**
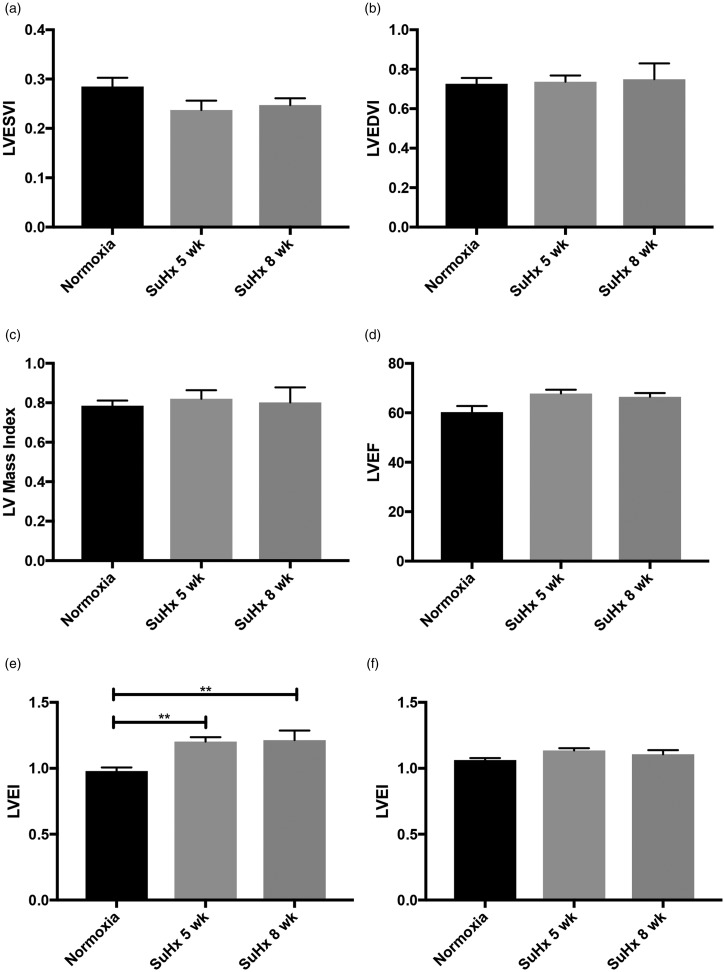
LV end-systolic volume index (LVESVI) (a), LV end-diastolic volume index (LVEDVI) (b), LV mass index (c), LV ejection fraction (LVEF) (d), LV eccentricity index (LVEI) in systole (e) and LV eccentricity index in diastole (f) are shown in normoxia (*n* = 8), Sugen–hypoxia at five weeks (*n* = 4) and Sugen–hypoxia at eight weeks (*n* = 4). LV end-diastolic and end-systolic volumes and LV mass were determined by manual planimetry and indexed to body surface area. LVEF was determined by ((LVEDV – LVESV)/LVEDV) × 100%. There were no significant differences between the normoxic group and different stages of Sugen–hypoxia in LVESVI, LVEDVI, LV mass index, and LVEF. Left ventricular eccentricity index (LVEI) was defined as the ratio of the anterior-inferior and septal–posterolateral cavity dimensions at the mid-ventricular level and was measured at both systole (e) and diastole (f). Although there were no significant differences between the three groups in the LVEI in diastole, LVEI was higher in both Sugen–hypoxic groups compared to normoxia in systole. Previous human studies had demonstrated LVEI in systole to correlate with pulmonary hypertension. The groups were compared by ANOVA, and if there was statistical significance, a Tukey HSD test was used for post hoc analysis. Results are shown as mean ± SEM. **: *p* < 0.05. LV: left ventricle; SuHx: Sugen–hypoxia.

No differences were observed between the normoxic and SuHx groups (normoxia, five and eight weeks, respectively) in terms of LVEDV index (0.73 ± 0.08 vs 0.74 ± 0.06 vs 0.75 ± 0.16), LVESV index (0.29 ± 0.05 vs 0.24 ± 0.04 vs 0.25 ± 0.03), LV mass index (0.79 ± 0.07 vs 0.82 ± 0.09 vs 0.8 ± 0.15), or LVEF (60.3 ± 7.03 vs 67.8 ±3.16 vs 66.5 ± 3.12) (Fig. 5).

### LV eccentricity index

LVEI measured at systole was significantly higher in SuHx rats at five weeks (1.2 ± 0.07, *p* = 0.006) and in SuHx rats at eight weeks (1.22 ± 0.14, *p* = 0.004) compared to normoxic rats (0.98 ± 0.08). There were no differences between LVEI at diastole between normoxia and SuHx at five or eight weeks (1.06 ± 0.05 vs 1.14 ± 0.04 vs 1.1 ± 0.06) (Fig. 5).

Supplementary file demonstrates CMR variables between normoxia and Sugen hypoxia at five and eight weeks.

### Autopsy vs CMR in the measurement of RV hypertrophy

**Fig. 6.**
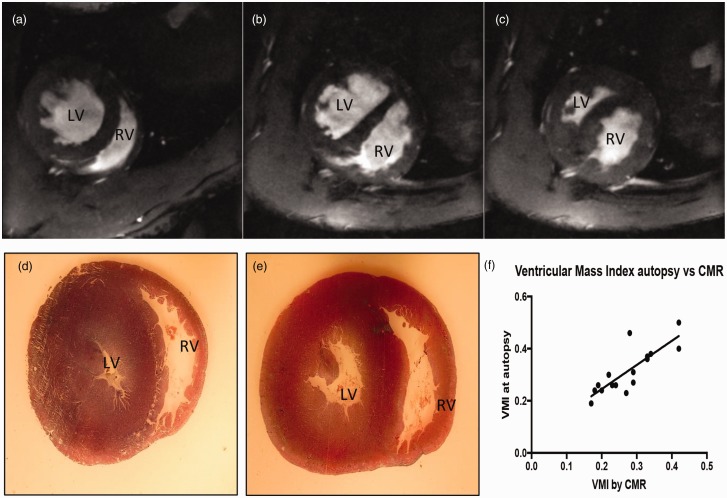
Cardiac MR images ((a) to (c)) and light microscopy images ((d) and (e)) of short axis sections of rat hearts. (a) and (d) demonstrate a normoxic rat. (b), (c), and (e) demonstrate a Sugen–hypoxic rat. (b) and (c) demonstrates the same animal short axis at diastole (b) and systole (c). (f) demonstrates the correlation between RV hypertrophy assessed by weighing RV and LV + S at autopsy and by ventricular mass index by CMR in normoxic rats and Sugen–hypoxic rats. There was very good correlation between VMI measured by CMR vs autopsy (Spearmen *r* = 0.8328). However, CMR images demonstrate functional aspects of RV contraction including septal flattening and paradoxical septal motion during systole (c) due to RV pressure overload. LV: left ventricle; RV: right ventricle; CMR: cardiac magnetic resonance; VMI: ventricular mass index.

CMR images taken from a normoxic and SuHx animals and light microscopy images of the same animals at autopsy were compared. Although both techniques could visually demonstrate ventricular size and wall thickness (hypertrophy), CMR demonstrated functional aspects of RV contraction including septal flattening and paradoxical septal motion during systole in SuHx animals. Fig. 6 demonstrates short axis CMR images ((a) to (c)) and light microscopy images ((d) and (e)) of short axis sections of the same rat hearts at autopsy. (b) and (c) demonstrates a SuHx animal at the same short axis at diastole (b) and systole (c). The SuHx animal demonstrated a significantly dilated and hypertrophied RV with paradoxical septal motion at systole. There was very good correlation between VMI measured by CMR vs autopsy (Spearmen *r* = 0.8328, 95% CI: 0.5633–0.9422, *p* < 0.0001). However, MR images demonstrate functional aspects of RV contraction including septal flattening and paradoxical septal motion during systole due to RV pressure overload.

## Discussion

To the best of our knowledge, this is the first study demonstrating longitudinal biventricular mass, volume, and function in a PH animal model using CMR. Previous studies had used echocardiography to examine biventricular function and structure in small animal models of PAH.^7^ Previous CMR studies used phase contrast imaging but did not explore LV and RV volume and functional variables known to be prognostic in PH, nor look at longitudinal changes.^10^

Our study demonstrated the feasibility of CMR in a small animal model of PAH with good spatial and temporal resolution and excellent inter-observer reproducibility. The future advantage of CMR over cardiac catheterization and autopsy is the ability to perform imaging serially on the same animal to look at disease progression and response to treatment without killing the animal.

The main cause of morbidity and mortality in patients with PAH is RV failure. It had been assumed that the cause of RV dysfunction was alterations to the pulmonary vasculature and therefore treatment focus had been centered on improving pulmonary hemodynamics, with the assumption that improvement in RV would follow. However, it is now evident that cardiac response to a given level of pulmonary hemodynamic overload is variable but important in the subsequent prognosis of these patients.^3^ Although traditionally right heart catheterization and post mortem studies have been used, there is a need for non-invasive tests of RV function in animal models of PAH. In addition, we need to have a better understanding of the longitudinal changes in ventricular function in animal models of PAH. Although the RV is the obvious focus of attention, LV dysfunction can occur through cardiac interaction, thus simultaneous evaluation of the LV is important. Echocardiography is widely available and can be used to estimate RVSP; however, imaging of the RV with its complex geometry is difficult.

### The SuHx small animal model—PH

Plexiform lesions in the pulmonary vasculature are known to be the hallmark of PAH and attempts have been made to establish an animal model that closely mimics human disease. The SuHx model has been shown to cause severe PH with the development of plexiform lesions. We used a SuHx model consisting of an injection of Sugen, two weeks of hypobaric hypoxia and three weeks or normoxia. Dean et al. explored the effects of Metformin on the development of PH via aromatase inhibition, a similar SuHx model was used (Sugen + two weeks of hypobaric hypoxia and three weeks of normoxia).^14^ The study demonstrated similar hemodynamics and RV hypertrophy compared to our study population after eight weeks. Abe et al. investigated the longitudinal hemodynamic and histological changes in the model. At 13–14 weeks after Sugen, the rats had very high RVSP (96 ± 11 vs 21) and severe RV hypertrophy (0.76 at five weeks, 0.74 at eight weeks, and 0.74 at 13–14 weeks).^6^ The varying degrees of RVSP and RV hypertrophy in these studies is likely related to the duration of exposure to hypoxia, however, could also be related to the animal strain, gender, and age at exposure.

### The SuHx small animal model—RV function

In humans with PAH, progressive narrowing of the pulmonary vasculature causes increased load to the RV. The RV adaptation results in increasing wall thickness (hypertrophy) and contractility (coupling). Ventriculo–arterial coupling preserves stroke volume and ventricular efficiency. The RV then dilates, increasing wall stress and oxygen consumption per gram resulting in uncoupling and reduced stroke volume.^15^ In our study, RV hypertrophy was followed by subsequent dilatation. However, stroke volume and RVEF were relatively preserved. This was despite the presence of persistently elevated RVSP. We believe this study represents the natural history of RV hypertrophy and failure, demonstrating compensated RV hypertrophy (adaptive remodeling) before progression into maladaptive remodeling with further RV dilatation and RV failure, with reduction of RV output.

### Adaptive vs maladaptive remodeling

Wang et al.^16^ hypothesized that a SuHx mouse model may capture the transition from adaptive to maladaptive RV remodeling including impairment in RV function by studying PV measurements in vivo. The results suggested that RV remodeling may begin to shift from adaptive to maladaptive with increasing duration of SuHx exposure. However, for the duration of SuHx exposure used in their study, no drop in cardiac output was observed.^16^ We believe that our study demonstrates a period of adaptive remodeling of the RV with compensated hypertrophy with minimal RV dilatation at later stages of the study. By lengthening the exposure to SuHx, we may be able to identify the transition from adaptive to maladaptive remodeling and identify decompensated right heart failure in this model. Most patients present to clinical assessment when there are signs of more severe RV dysfunction and in contrast pre-clinical SuHx model demonstrate adaptive remodeling at least early in its disease progression. This is of importance in the design of pre-clinical studies as intervention with experimental therapeutics is likely to occur at this early adaptive stage of disease progression where RV function/stroke volume seem to be preserved despite the presence of PAH.

### Small animal model—LV function

In PAH, impaired LV performance is explained by low RV cardiac output and direct ventricular interaction due to inter-ventricular dyssynchrony and paradoxical septal motion.^17,18^ Previous human studies have demonstrated that patients with progressive illness demonstrated lower LV systolic function compared to stable patients. Previous echocardiography studies have also demonstrated impaired LV strain and torsion in PAH patients.^17^ We observed preserved LV systolic function with preserved LV mass and volume variables in this small animal model of PAH. Although PAH animals demonstrated paradoxical septal motion of the septum in our study, preserved RV output probably explains the preserved LV function.

### Conclusion

CMR is feasible in a small animal model of PAH and could be used in pre-clinical animal studies to explore bi-ventricular structural and functional changes during the course of illness. It is likely that the SuHx model demonstrates adaptive remodeling to persistently elevated pulmonary pressures which is demonstrable by preserved RV function and stroke volume with hypertrophy of the RV. Further longitudinal studies are required to assess this model in detail, especially focusing on longitudinal RV response. The search for better animal models of PH continues because our understanding of the pathobiology of disease and the development of new therapeutic strategies depends on robust animal models, but at present, no single model has all the features of human disease.

### Limitations

The study assessed rats at five and eight weeks from Sugen exposure, while assessment later may have demonstrated worsened hemodynamics and RV variables mimicking disease presentation in humans. The rats were anesthetized with isoflurane, previous studies using halogenated anesthetics have been shown to impair RV–PA coupling; however, these effects were seen both in hypoxia and hyperoxia.^19^ Future studies with late gadolinium enhancement data will provide further insights into myocardial remodeling in PAH.

## Supplemental Material

PUL897513 Supplemental material - Supplemental material for Understanding longitudinal biventricular structural and functional changes in a pulmonary hypertension Sugen–hypoxia rat model by cardiac magnetic resonance imaging Click here for additional data file. Supplemental material, PUL897513 Supplemental material for Understanding longitudinal biventricular structural and functional changes in a pulmonary hypertension Sugen–hypoxia rat model by cardiac magnetic resonance imaging by Geeshath Jayasekera, Kathryn S. Wilson, Hanna Buist, Rosemary Woodward, Aysel Uckan, Colin Hughes, Margaret Nilsen, A Colin Church, Martin K. Johnson, Lindsay Gallagher, James Mullin, Mandy R. MacLean, William M. Holmes, Andrew J. Peacock and David J. Welsh in Pulmonary Circulation
