# Supplementary material for: Understanding longitudinal biventricular structural and functional changes in a pulmonary hypertension Sugen–hypoxia rat model by cardiac magnetic resonance imaging
Source: Pulm Circ. 2020 Feb 10;10(1):2045894019897513. doi: 10.1177/2045894019897513 (PMC7011361; doi:10.1177/2045894019897513)
Supplement: PUL897513 Supplemental material - Supplemental material for Understanding longitudinal biventricular structural and functional changes in a pulmonary hypertension Sugen–hypoxia rat model by cardiac magnetic resonance imaging [file PUL897513_Supplemental_material.pdf]

	Normoxia	***SuHx 5 wk***	***SuHx 8 wk***
Weight (g)	411.4 ± 50.8 (368.9 - 453.9)	311.3 ± 8.5 (297.8 - 324.7)	424.3 ± 55.7 (335.7 - 512. 8)
LVEF	60.3 ± 7 (54.4 - 66.2)	67.8 ± 3.2 (62.8 - 72.8)	66.5 ± 3.1 (61.5 - 71.5)
LVEDV	393.7 ± 50.8 (351.3 - 436.2)	332.6 ± 28.7 (286.9 - 378.3)	410.7 ± 63 (310.4 - 510.9)
LVEDVI	0.73 ± 0.08 (0.66 - 0.8)	0.74 ± 0.06 (0.64 - 0.84)	0.75 ± 0.16 (0.5 – 1)
LVESV	155.9 ± 33.8 (127.6 - 184.1)	107.4 ± 16.8 (80.6 - 134.1)	136.3 ± 11.6 (117.8 - 154.8)
LVESVI	0.29 ± 0.05 (0.24 - 0.33)	0.24 ± 0.04 (0.18 - 0.3)	0.25 ± 0.03 (0.2 - 0.29)
SV (LV)	237.8 ± 44.8 (200.4 - 275.3)	225.3 ± 17.3 (197.8 - 252.7)	274.3 ± 53.5 (189.2 - 359.4)
SVI	0.44 ± 0.1 (0.37 - 0.51)	0.5 ± 0.04 (0.45 - 0.55)	0.49 ± 0.1 (0.3 - 0.69)
LV mass	426.6 ± 55 (380.6 - 472.6)	371.1 ± 46 (298 - 444.3)	440.4 ± 48.4 (363.4 - 517.4)
LV mass index	0.79 ± 0.07 (0.72 - 0.84)	0.82 ± 0.09 (0.68 - 0.96)	0.8 ± 0.15 (0.56 - 1.04)
RVEF	68.3 ± 5.1 (64.1 - 72.5)	69.4 ± 6.9 (58.4 - 80.3)	62.6 ± 6.1 (52.9 - 72.3)
RVEDV	303.5 ± 49.4 (262.3 - 344.8)	316.6 ± 16.6 (290.2 - 343)	409.1 ± 46.8 (334.6 - 483.7)
RVEDVI	0.56 ± 0.1 (0.48 - 0.64)	0.7 ± 0.03 (0.66 - 0.75)	0.75 ± 0.13 (0.54 - 0.95)
RVESV	95.7 ± 18.6 (80.1 - 111.2)	97.5 ± 25.3 (57.2 - 137.7)	151.8 ± 19.1 (121.4 - 182.2)
RVESVI	0.18 ± 0.03 (0.15 - 0.19)	0.22 ± 0.06 (0.12 - 0.31)	0.28 ± 0.04 (0.21 - 0.34)
RV mass	91.2 ± 20.6 (73. 9 - 108.4)	123.3 ± 19.1 (93 - 153.7)	148 ± 14 (125.8 - 170.3)
RV mass index	0.17 ± 0.03 (0.14 - 0.2)	0.28 ± 0.04 (0.21 - 0.34)	0.27 ± 0.04 (0.2 -0.34)
Ventricular mass index (VMI)	0.21 ± 0.04 (0.18 - 0.24)	0.34 ± 0.06 (0.24 - 0.43)	0.34 ± 0.06 (0.25 - 0.43)
LVEI at systole	0.98 ± 0.08 (0.91 - 1.05)	1.2 ± 0.07 (1.09 - 1.31)	1.22 ± 0.14 (0.99 - 1.45)
LVEI at diastole	1.06 ± 0.05 (1.02 -1.1)	1.14 ± 0.04 (1.1 -1.19)	1.1 ± 0.06 (1.01 - 1.2)

Supplementary file. Cardiac MRI variables between normoxia, 5 week Sugen hypoxia (SuHx 5 wk) and 8 week Sugen hypoxia (SuHx 8 wk) are demonstrated. Ventricular volumes and mass are given as µl and mg, and as µlcm^{-2} and mgcm^{-2} respectively when indexed for body surface area. LV ejection fraction (LVEF), LV end diastolic volume (LVEDV), LV end diastolic volume index (LVEDVI), LV end systolic volume (LVESV), LV end systolic volume index (LVESVI), stroke volume (SV), stroke volume index (SVI), RV ejection fraction (RVEF), RV end diastolic volume (RVEDV), RV end diastolic volume index (RVEDVI), RV end systolic volume (RVESV), RV end systolic volume index (RVESVI), LV eccentricity index (LVEI) at systole and diastole are shown. All values are shown as mean ± standard deviation (95 % confidence interval).
